# Japanese encephalitis virus NS1 inhibits IFN-β production by interacting with DDX3X

**DOI:** 10.1128/jvi.00077-25

**Published:** 2025-04-15

**Authors:** Hao Dong, Yue Hao, Jue Wang, Dengjin Chen, Shengkui Xu, Wenke Ruan

**Affiliations:** 1 Beijing Key Laboratory of Traditional Chinese Veterinary Medicine, College of Veterinary Medicine, Beijing University of Agriculture 74684, Changping, Beijing, China; 2 China Animal Husbandry Industry Co. Ltd., Beijing, China; University of Michigan Medical School, Ann Arbor, Michigan, USA

**Keywords:** Japanese encephalitis virus (JEV), non-structural protein 1 (NS1), DDX3X, IFN-β, ISGs

## Abstract

Japanese encephalitis virus (JEV) is the causative agent of Japanese encephalitis, which poses great threats to the pig farming industry and human health. To establish infection, JEV has evolved sophisticated strategies to overcome the innate immune responses and finish its life cycle. Previous studies have shown that non-structural protein 1 (NS1) is closely related to its pathogenesis, while its molecular mechanism remains elusive. In this study, host protein ATP-dependent RNA helicase DEAD-box helicase 3 X (DDX3X) was screened to bind with NS1, and both ectopically expressed and virally encoded NS1 further confirmed their interaction. We also proved that the β-roll and wing subdomains of NS1 were responsible for their interaction. In DDX3X-overexpressing cells, the replication of JEV was markedly inhibited, while the viral titers were elevated in DDX3X-silencing cells, indicating that DDX3X might serve as an anti-viral factor during JEV infection. Mechanically, overexpression of DDX3X promotes interferon-beta (IFN-β) transcription, while its transcription was decreased in DDX3X-silencing cells. Consistent with IFN-β, some interferon-stimulated genes (ISGs), including protein kinase R (PKR), myxovirus resistance 1 (MX1), guanylate-binding protein 1 (GBP1), and bone marrow stromal antigen 2 (BST2), were also positively related to the DDX3X expression. Taken together, JEV NS1 blocks IFN-β production by interacting with DDX3X to evade the host’s innate immune response and facilitate virus replication. This finding will deepen our understanding of JEV immune-evasion strategies and provide targets for JEV attenuation.

**IMPORTANCE:**

This study focused on JEV, a threat to pig farming and human health. The key finding is that NS1 binds to host protein DDX3X via its β-roll and wing subdomains. JEV NS1 evades the host immune response by interacting with DDX3X to restrain type I interferon production. These results deepen our understanding of JEV’s immune-evasion strategies and offer potential targets for JEV attenuation.

## INTRODUCTION

Japanese encephalitis virus (JEV) is the causative agent of Japanese encephalitis, which is characterized by neurologic complications and reproductive disorders in pigs (1, 2). The first case was reported in Japan in 1871, while the causative pathogen was successfully isolated 60 years later (3). Since then, JEV cases have been reported in many countries, including South Korea, China, the Philippines, and India (4). The World Health Organization has identified JEV as endemic in about 24 countries across Southeast Asia and the Western Pacific, putting more than 3 billion people at risk of infection. JEV has a wide host range, including humans, birds, pigs, and mosquitoes. Pigs infected with JEV may present clinical symptoms such as abortion, stillbirth, and boar orchitis during pregnancy (5). In humans, JEV infection may cause high fever, headache, encephalitis, and even death in severe cases (6). JEV, a member of the *Orthoflavivirus* genus, possesses a genome approximately 11 kb in length. The genome comprises a single open reading frame (ORF) flanked by 5′ and 3′ untranslated regions (UTRs) (7). The only ORF is translated into one polyprotein precursor, which is then cleaved into three structural proteins (capsid protein [C], membrane protein [M], and envelope protein [E]) and seven non-structural proteins (non-structural protein 1 [NS1], NS2A, NS2B, NS3, NS4A, NS4B, and NS5) (7).

Once entering the cells, the virus replicates its genomes in the replication-transcription complexes formed in the endoplasmic reticulum. The viral genomes and replicative intermediates of double-stranded RNA can be sensed by pattern recognition receptors (PRRs) and activate the innate immune response. As powerful activators of the innate immune response, viral components initiate a series of signaling cascades that boost the production of inflammatory factors and type I interferon (8, 9). During this process, non-structural proteins of JEV were involved in virus replication and innate immunity modulation. For example, NS1 exists as either a dimer or a hexamer, with each monomer comprising three distinct domains: a hydrophobic β-roll (amino acids 1–29), a wing domain (amino acids 30–180), and a β-ladder domain. These domains are interconnected by disulfide bonds and serve as a key immunogen during viral infection (10). Comparative analysis of NS1 in West Nile virus (WNV), yellow fever virus, and dengue virus (DENV) revealed that NS1 interacts with a variety of host proteins to promote viral replication (11). NS1 suppresses the Janus kinase-signal transducer and activator of transcription (JAK-STAT) signaling pathway in the midguts of mosquitoes, thereby enhancing the acquisition of viruses by these vectors (12). Additionally, NS1 activates innate immunity through TLR4 during DENV infection, while it suppresses NF-κB nuclear translocation during WNV infection (13). Studies have shown that NS1 interacted with vimentin and heterogeneous nuclear ribonucleoprotein K to promote viral replication during JEV infection (14). NS1′, produced in NS1 by ribosome frameshift, interacts with host cyclin-dependent kinase 1 protein to inhibit cellular anti-viral responses and promote JEV replication (15). In addition, NS1′ antagonizes host innate immune factor mitochondrial anti-viral signaling (MAVS) protein to inhibit interferon-beta (IFN-β) production and promote viral replication (16). Therefore, further elucidation of the interaction between JEV NS1 and host cell proteins will improve our understanding of its innate immune regulation capacity.

Innate immunity is the primary defense line against virus infections. Once activated, PRRs recruit the downstream signaling mediators and trigger the production of interferon (IFN), inflammatory factors, and chemokines. IFNs bind to their receptor and activate the JAK-STAT pathway to induce the production of various interferon-stimulated genes (ISGs) and finally establish an anti-viral state in host cells to limit the spread of viruses. DDX3X, belonging to the DExD-box helicase family, contains nine conserved motifs with RNA-dependent ATPase and ATP-dependent RNA unwinding activity (17). Various reports have proven that DDX3X participates in mRNA transcription, metabolism, protein translation, and anti-viral responses (18). Correspondingly, viruses may manipulate DExD/H-box proteins to regulate the immune system and facilitate their replication (19). Oshiumi et al. reported that DDX3X interacted with the MAVS to boost the innate immune responses (20). Moreover, vaccinia virus-encoded protein K7 prevents DDX3 from inducing the IFN-β promoter by binding to the N-terminus of DDX3; the polymerase protein pol encoded by the hepatitis B virus inhibits the activities of TANK-binding kinase 1 and inhibitor of nuclear factor kappa-B kinase epsilon (IKKε) by disrupting the interaction between DDX3 and IKKε, thereby effectively antagonizing the host’s innate immune response (21). As for flavivirus, DDX3X can stimulate the transcription of IFN-β through the IRF3 and NF-κB pathways during dengue virus infection to hinder virus replication (22); HCV core protein sequesters DDX3X in the stress granules to abrogate the anti-viral function of DDX3 (23).

In regard to the characteristics of JEV non-structural proteins and DDX3X in innate immune modulation, we aim to clarify the effects of NS1 and DDX3X on JEV replication. In this study, we identified the NS1-DDX3X interaction and evaluated their biological significance. The results not only deepen our understanding of how JEV evades innate immune clearance but also provide clues for virus attenuation.

## MATERIALS AND METHODS

### Cell and virus

BHK-21 cells were purchased from the American Type Culture Collection and maintained in Dulbecco's modified Eagle medium (DMEM) supplemented with 10% fetal bovine serum (FBS) and penicillin (100 U/mL) at 37°C in 5% CO_2_. The SA14-14-2 strain (GenBank accession number MK585066.1) of JEV was stored in our lab and propagated in BHK-21 cells.

### Antibodies

The primary antibodies were specific for DDX3X (ab196032, Abcam), rabbit anti-phospho-DDX3 (Thr322) antibody (bs-12190R; Bioss, Beijing), β-actin (TA-09; ZSGB-Bio, Beijing), glyceraldehyde 3-phosphate dehydrogenase (GAPDH) (TA-08, ZSGB-Bio), GST (TA-03, ZSGB-Bio), and His (TA-02, ZSGB-Bio). Anti-mouse (A0216) and anti-rabbit (A0208) horseradish peroxidase-conjugated secondary antibodies were purchased from Beyotime Company (Beijing, China). Antibodies against NS1 and E were prepared in this study.

### Liquid chromatography-tandem mass spectrometry

Liquid chromatography-tandem mass spectrometry analysis was carried out with Thermo’s Q Exactive Plus system. Sample separation was achieved through the liquid-phase UltiMate 3000 RSLCnano system. Subsequently, ProteinPilot (v.4.5) was utilized to extract the mass spectrum data generated by Q Exactive Plus, with reference to the Gallus database. Comparative analysis of proteins was then conducted, during which specific peptide segments were matched by the database and scored.

### Plasmid transfection

BHK-21 cells were seeded into six-well cell plates for 12 h. Four micrograms pEGFP-NS1-FL and the truncated forms of pEGFP-NS1-FL, pMyc-DDX3X, pFlag-DDX3X, and siRNA-DDX3X (sequence: GCCCAACUCCAGUACAGAATT) were transfected using jetPRIME (101000046, Ployplus) according to the manufacturer’s instructions when the cell density reached 70%–80%. The whole cell lysate was collected at 48 h and subjected to Western blot or co-immunoprecipitation (Co-IP) analysis.

### Protein expression and purification

To determine NS1-DDX3X interaction *in vitro*, the DDX3X and NS1 proteins were expressed and purified in *Escherichia coli*. pGEX-DDX3X and pET-28a-NS1 were transformed into the *E. coli* strain BL21 (DE3) and cultivated in Luria-Bertani medium containing ampicillin (100 µg/mL) or kanamycin (50 µg/mL). When the OD_600_ reached 0.6–0.8, 1 mM isopropyl β-D-1-thiogalactopyranoside (IPTG) was added to induce protein expression for 12 h at 18°C. Upon harvesting by centrifugation, the cells were resuspended in a lysis buffer (100 mM NaCl, 20 mM Tris-HCl, pH 8.0, 0.1 mM phenylmethylsulfonyl fluoride) and disrupted by sonication. The DDX3X and NS1 protein in the supernatant were purified by affinity chromatography assay using UniNTA-80Ni (Nano Micro Tech, Suzhou, China) or GST Focurose 4FF (Huiyan Biotech, Wuhan, China).

### GST pull-down assay

The GST pull-down assay was conducted as follows: the BeyoMag GST beads (P2258, Beyotime Company) were mixed with purified GST-DDX3X and His-NS1-FL with binding buffer followed by incubation at room temperature for 60 min; the beads were separated on a magnetic stand for 10 seconds to remove the supernatant; the GST-purified agarose magnetic beads were resuspended gently by adding 2 mL wash buffer; the beads were separated on a magnetic stand for 10 seconds to remove the supernatant; steps 2–4 were then replicated three times; 0.2 mL elution buffer was added; the tube was rotated gently for 5 min, then the magnetic beads were separated and the supernatants were collected; the collected proteins were subjected to Western blot to detect NS1-DDX3X interaction.

### Western blot

Cell lysis buffer (R0100, Solarbio) containing a protease inhibitor cocktail (87785, Thermo Fisher) was used to lyse the cells, and the whole protein concentrations were measured by the BCA Protein Quantification Kit (E112-01, Vazyme). Twenty micrograms of protein was subjected to SDS-PAGE and transferred to polyvinylidene fluoride membranes (88518, Thermo Fisher). The membranes were blocked with 5% skim milk and incubated with the indicated primary antibodies at 4°C overnight. Tris-buffered saline with Tween-20 (0.1% Tween-20) was used to wash the membranes, followed by incubation of the secondary antibodies for 1 h at room temperature. Membrane imaging was performed using ECL (32209, Thermo Fisher).

### Co-IP assay

The BHK-21 cells were washed twice with pre-cold phosphate-buffered saline (PBS). Afterward, the cells were incubated in lysis buffer for 10–20 min at 4°C and centrifuged to remove debris. Protein A/G agarose beads coupled to anti-DDX3, EGFP, or NS1 polyclonal antibodies were co-incubated with the processed samples for 2 h at 4°C. The samples were washed three times and resuspended in 5× sample buffer before being subjected to Western blot analysis. The proteins were then detected with antibodies against DDX3X, NS1, and EGFP.

### Quantitative reverse transcription PCR

The total RNA was extracted using VeZol reagent (R411-01, Vazyme) according to the manufacturer’s protocol and measured by NanoDrop. One microgram of total RNA was reverse-transcribed randomly to generate cDNA with the miRNA 1st Strand cDNA Synthesis Kit (MR101-01, Vazyme). All quantitative reverse transcription PCR (qRT-PCR) was performed using the SYBR Green master mix (Q111-02, Vazyme) on a Quant Studio3 Real-Time PCR system (Applied Biosystems). The housekeeping gene GAPDH was used as a reference gene. All qRT-PCR assays were carried out for three independent replicates. Then, the 2^−ΔΔCt^ method was applied to quantify the relative mRNA expression level. Primer sequences are listed in Table 1.

**TABLE 1:** qRT-PCR primers used in the study

Primer name	Sequence of oligonucleotide (F/R) (5′−3′)
GAPDH-PF/PR	TCGTGGATCTCACATGTCGC/TGAAGTCGCAGGAGACAACC
IFNβ-PF/PR	CTTGGCTTTGTTGCTCTGCC/AGCAAACAGGTCTGCCTTGA
ISG15-PF/PR	TCTATGAGGTCCGGCTGACA/GCACTGGGGCTTTAGGTCAT
OAS1-PF/PR	GAAAAGGCCTATGCCTGGGT/TTTACGGAAGGTGGTGTCGG
PKR-PF/PR	AGCTCAATGAATGGGCTCAGA/ACTTGGCCAAATCCACCTGA
ISG20-PF/PR	CACCCCCTCAGAACAACTGG/CTGTGGAAAATGACTGTCCTTGAG
MX1-PF/PR	ACTGTTACCAGAGTGCGAGC/GGATCTTGGTGAACAGGCGA
GBP1-PF/PR	CAAAAGCCGACAGGAGACGA/TGCGGTAGAGACCCACAATG
IFI27-PF/PR	GATGTCAGCAGCAGCAATCG/AGTTCAAAGCAAGGCTCCGT
CD47-PF/PR	TGAAAGGCGTTGCCTCTTTG/GAAAACCATGACGCCACACG
ZC3H11A-PF/PR	GACAGGTGTGCAGATTTCGC/TCAAAACCAATCTCACTTTTGCT

### Plaque assay

The plaque assay is the most quantitative biological assay for viruses, and it was applied to measure JEV titers in BHK-21 cells. The specific steps are as follows: dilute the virus stocks 10-fold serially in 1.5 mL tubes and inoculate them into six-well plates for 2 h; discard the medium and rinse the cells with PBS twice; add 2 mL covering medium (2% low melting-point agarose with DMEM medium containing 2% FBS) gently to the plates and culture the cells at 37°C, in a 5% CO_2_ incubator for another 72 h; add 1 mL 4% paraformaldehyde containing crystal violet and incubate for 30 min; discard the crystal violet and wash the plates with PBS three times. The number of plaques was counted using Image J software.

### Statistical analysis

GraphPad Prism (v.9.0) software was used for data analysis. Statistical significance was determined by unpaired two-tailed *t*-test and one-way and two-way analyses of variance. Every experiment was conducted for at least three biologically independent replicates. * indicates *P* < 0.05; ** indicates *P* < 0.01; *** indicates *P* < 0.001; and **** indicates *P* ≤ 0.0001. All data supporting the findings of this study are available within the paper.

## RESULTS

### Screening of host proteins interacting with JEV NS1

**Fig 1:**
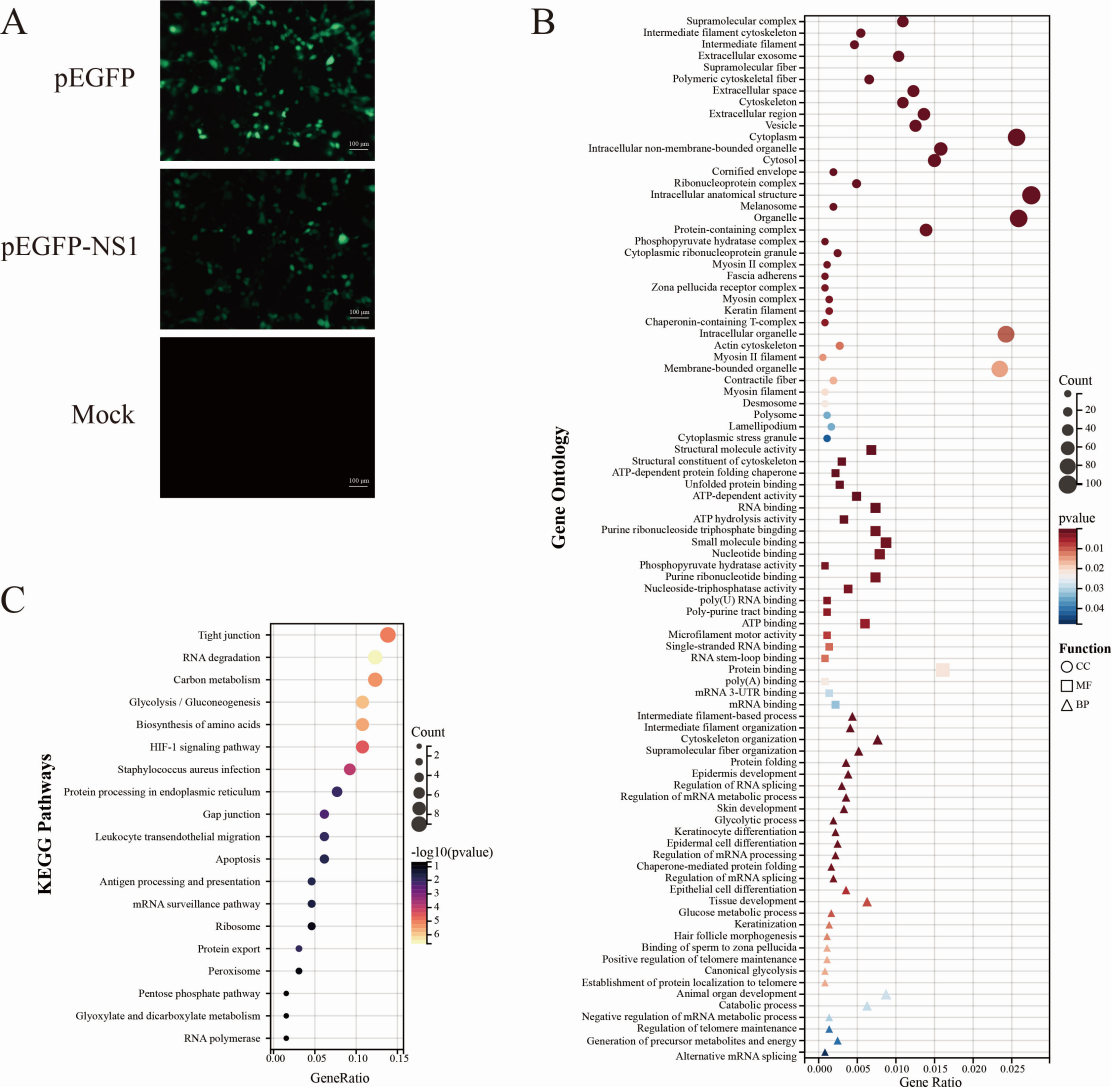
Bioinformatic analysis of NS1-interacting proteins (**A**) The pEGFP-NS1 was transfected into BHK-21 cells, and the fluorescence intensity of the transfected groups was observed 24 h later to identify NS1 expression. Scale bar = 100 µm. Biological functional enrichment analyses were performed on the enriched proteins, including Gene Ontology annotation (**B**) and Kyoto Encyclopedia of Genes and Genomes (KEGG) pathway analysis (**C**).

It is reported that NS1 of JEV could provide high immune protection *in vivo* (24). To explore its functions in regulating the host cell biological process, we first screened for host proteins interacting with NS1. JEV *NS1* was cloned into pEGFP-N1, and the obtained chimeric plasmid was designated with pEGFP-NS1. The pEGFP-NS1 was transfected into BHK-21 cells for 48 h, and the whole cell lysate was collected and subjected to Co-IP and mass spectrometry (MS) assays to screen NS1-binding proteins (Fig. 1A). The enriched proteins were subjected to Gene Ontology analysis, and the results showed that NS1-captured host proteins are mostly located in the cytoplasm and involved in protein binding (Fig. 1B). This indicates that NS1 might exert its functions in the cytosol by binding with various host proteins. Furthermore, Kyoto Encyclopedia of Genes and Genomes pathway analysis showed that the enriched proteins were mainly concentrated in tight junctions and RNA degradation pathways (Fig. 1C).

**Fig 2:**
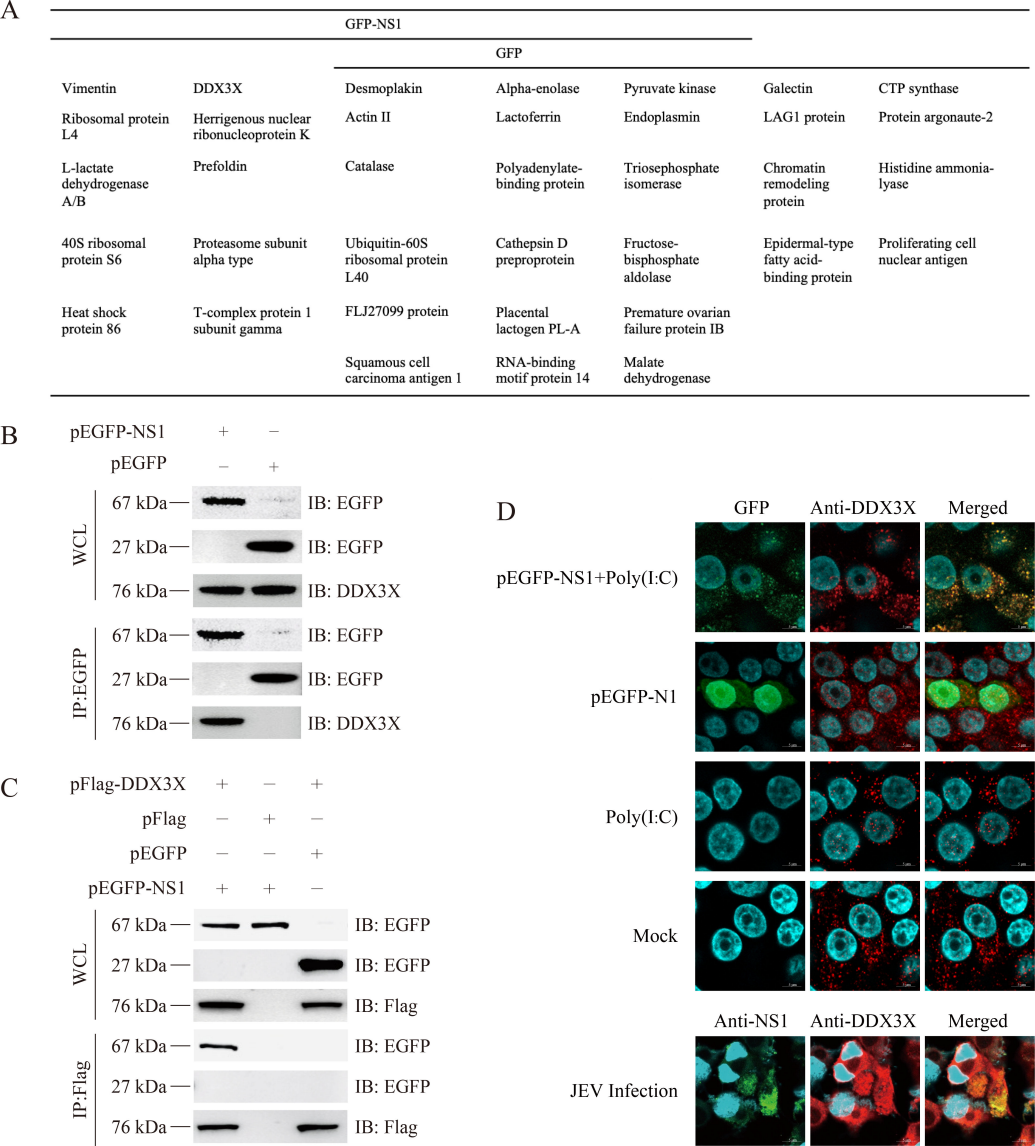
Screening and validation of host proteins interacting with NS1. (**A**) The list of NS1 interacting host proteins identified by liquid chromatography-tandem mass spectrometry. (**B**) BHK-21 cells were transfected with pEGFP-NS1 for 48 h, and then the whole cell lysates were subjected to Co-IP assay with antibody against EGFP. (**C**) BHK-21 cells were co-transfected with pFlag-DDX3X and pEGFP-NS1 for 48 h; the cell lysates were subjected to Co-IP assay with antibody against Flag. (**D**) BHK-21 cells were transfected or infected with pEGFP-N1, pEGFP-NS1, and JEV (0.1 multiplicity of infection), respectively, for 48 h, and immunofluorescence assay was applied to observe NS1-DDX3X interaction. Bars = 5 µm.

Based on the MS scores, 10 proteins specifically interacting with fusion protein GFP-NS1 but not GFP (14), including vimentin, heat shock protein 86, and DDX3X, were identified (Fig. 2A; Table S1). Considering its various functions in regulating innate immune response, DDX3X was selected for further study. To validate the interaction between DDX3X and NS1, pEGFP-NS1 was transfected into BHK-21 cells and then subjected to Co-IP analysis. The Western blot result showed that NS1 interacted with host cell DDX3X (Fig. 2B). In addition, DDX3X-NS1 interaction was further confirmed in both NS1 and DDX3X-overexpressing cells by Co-IP assay (Fig. 2C). Finally, the NS1-DDX3X interaction was confirmed through immunofluorescence assay. The images revealed that NS1 exhibited a dispersed distribution in the cytoplasm. Co-localization of NS1 and DDX3X (indicated by the yellow signal) was observed only when cells were infected with JEV or treated with poly(I:C) to mimic viral replication (Fig. 2D). In summary, NS1 interacts with both endogenous DDX3X and ectopically expressed DDX3X.

### NS1 interacts with DDX3X during virus infection

**Fig 3:**
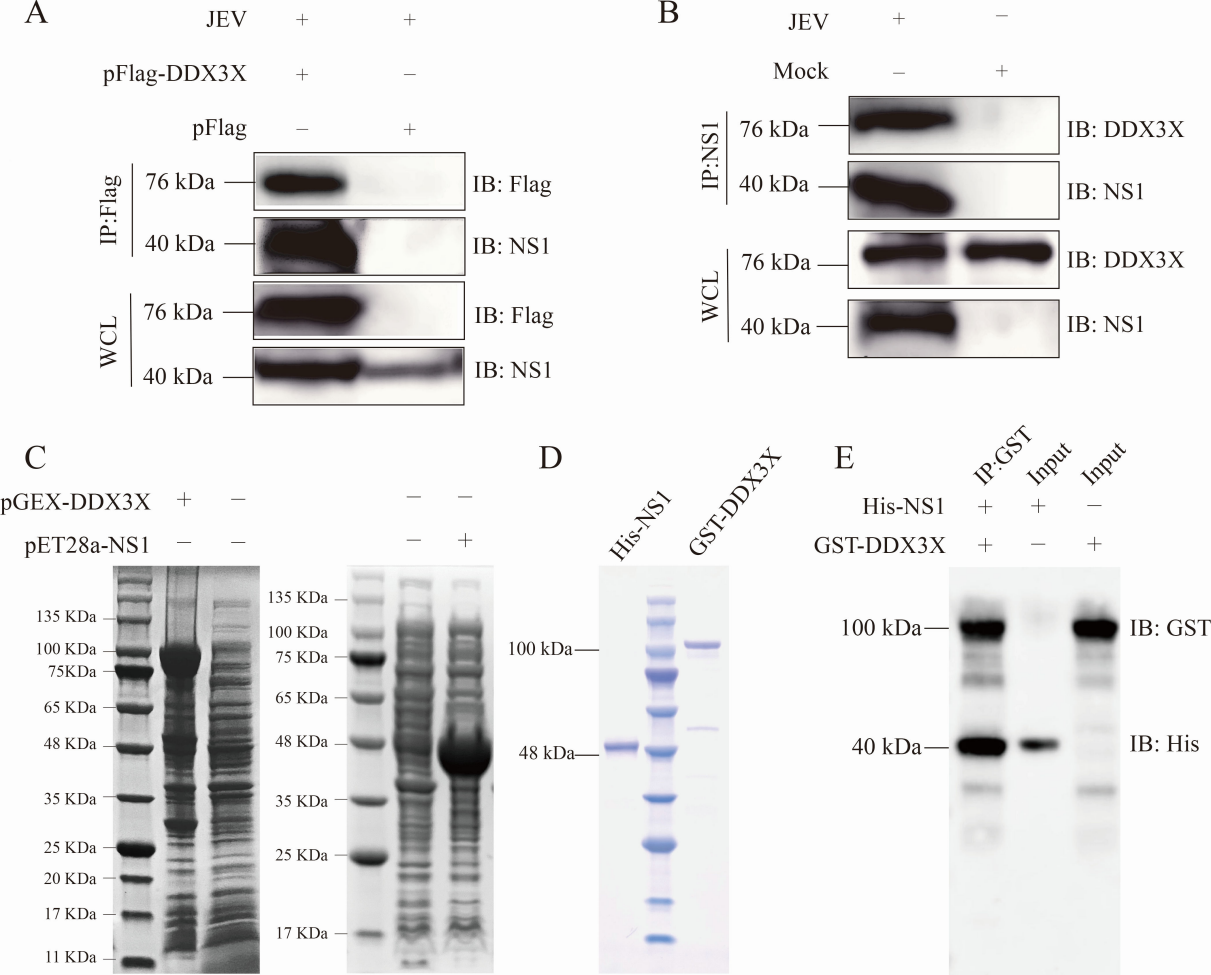
Verification of NS1-DDX3X interaction. (**A**) BHK-21 cells were transfected with pFlag-DDX3X for 24 h and then infected with JEV for another 24 h. The cell lysates were subjected to Co-IP with an antibody against Flag and detected by Western blot. (**B**) BHK-21 cells were infected with JEV for 48 h, and the whole cell lysates were subjected to Co-IP with an antibody against NS1. The endogenous DDX3X was detected with an antibody against DDX3X. (**C**) pGEX-DDX3X and pET28a-NS1 were respectively transformed into BL21 (DE3) to prepare protein. (**D**) The His-tagged NS1 and GST-tagged DDX3X were purified by the affinity chromatography method. (**E**) The His-tagged NS1 and GST-tagged DDX3X were co-incubated with anti-GST magnetic beads, and then the NS1 and DDX3X were detected by Western blot analysis with antibodies against GST and His tag.

To determine the interaction between NS1 and DDX3X during virus infection, BHK-21 cells were transfected with pFlag-DDX3X for 24 h and then infected with JEV at a multiplicity of infection (MOI) of 1 for another 24 h. The whole cell protein was collected and subjected to Co-IP assay. Antibody against Flag was used to precipitate JEV-encoded NS1, and Western blot results demonstrated that Flag-tagged DDX3X could co-immunoprecipitate with JEV-encoded NS1 during virus infection (Fig. 3A). Then, the interaction between endogenous DDX3X and JEV-encoded NS1 was further studied by the Co-IP assay. BHK-21 cells were infected with JEV (MOI = 1) for 48 h, and the whole cell lysates were collected and subjected to Co-IP with antibody against NS1. The Western blot results showed that the NS1 antibody could precipitate the endogenous DDX3X (Fig. 3B). Taken together, NS1 interacts with both endogenous and exogenous DDX3X.

To analyze if other host factors are involved in the NS1-DDX3X interaction, we expressed and purified His-tagged NS1 (40.3 kDa) and GST-tagged DDX3X (100.1 kDa), respectively, in *E. coli* (Fig. 3C and D). Then, the GST-pull-down result proved that NS1 could bind to DDX3X *in vitro* (Fig. 3E). The results showed that NS1 could bind to DDX3X directly in BHK-21 cells.

### Identification of critical domains of NS1 responsible for DDX3X interaction

**Fig 4:**
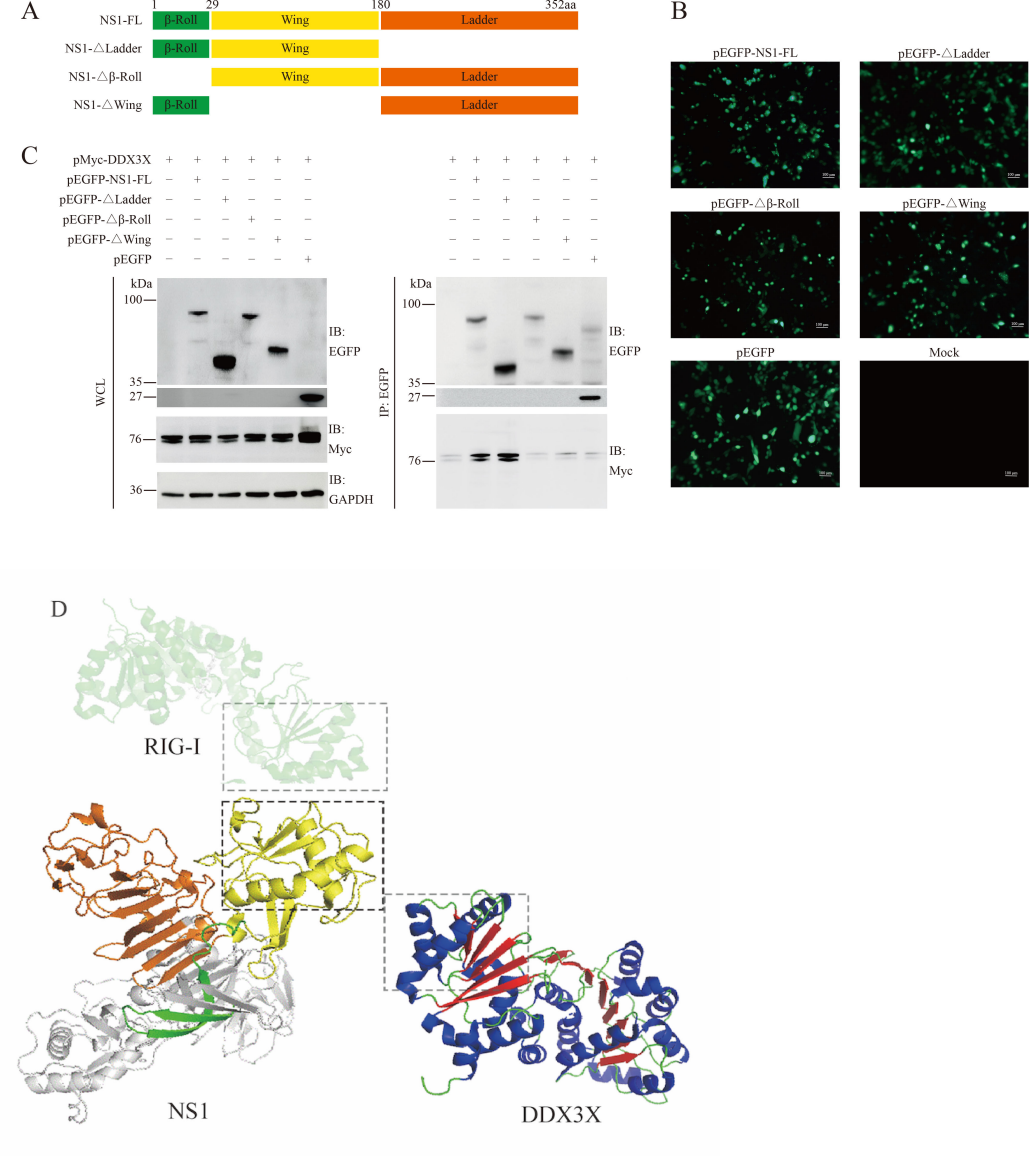
Screening of NS1 domains responsible for DDX3X interaction. (**A**) The NS1 protein (QCZ42158.1, with NS1 spanning positions 795–1,146) domains predicated using the protein structure prediction website and the schematic diagram of NS1 and its mutants. (**B**) The expression of NS1 mutants in BHK-21 cells identified by fluorescence assay. Bars = 100 µm. (**C**) The NS1 mutant plasmids and pMyc-DDX3X were co-transfected into BHK-21 cells for 48 h, and the whole cell lysates were subjected to Co-IP assay to determine their interaction. (**D**) The 3D structural similarity comparison of *Orthoflavivirus* NS1, RIG-I, and DDX3X.

NS1 is composed of three central regions: the β-roll, wing, and ladder (https://zhanggroup.org/research/#StructurePrediction). To further determine the critical domains of NS1 responsible for DDX3X binding, a series of NS1 truncated mutants were constructed according to NS1 domains (Fig. 4A). The NS1 truncated mutants were expressed in BHK-21 cells, as indicated by the fluorescence assay in Fig. 4B. To determine the interacting regions of NS1 more precisely, the pEGFP-NS1-FL, pEGFP-NS1-Δladder, pEGFP-NS1-Δβ-roll, pEGFP-NS1-Δwing were co-transfected with pMyc-DDX3X, respectively, for 48 h, and the whole cell lysis was subjected to Co-IP analysis. As shown in Fig. 4C, NS1-FL or NS1-Δladder, but not NS1-Δβ-roll and NS1-Δwing, could interact with DDX3X, indicating that amino acids 1–180 of NS1 were critical for DDX3X binding. NS1 was highly conserved in *Orthoflavivirus*, and the NS1 wing α/β subdomain resembles a helicase domain of innate immune protein retinoic acid-inducible gene I (RIG-I) (10) and DDX3X (Fig. 4D), which corresponds with the critical wing domain mediating DDX3X interaction (PDB IDs: DDX3X, 2I4I; RIG-I, 3TBK; NS1, 5GS6). To further study the effects of the two domains on virus replication, we attempted to rescue JEV-ΔRW (a mutant JEV with NS1 lacking the β-roll and wing domains). Regrettably, the rescue of the mutant virus was unsuccessful (see Fig. 6D). This result strongly suggests that the β-roll and wing domains are indispensable for the completion of the viral life cycle.

**Fig 5:**
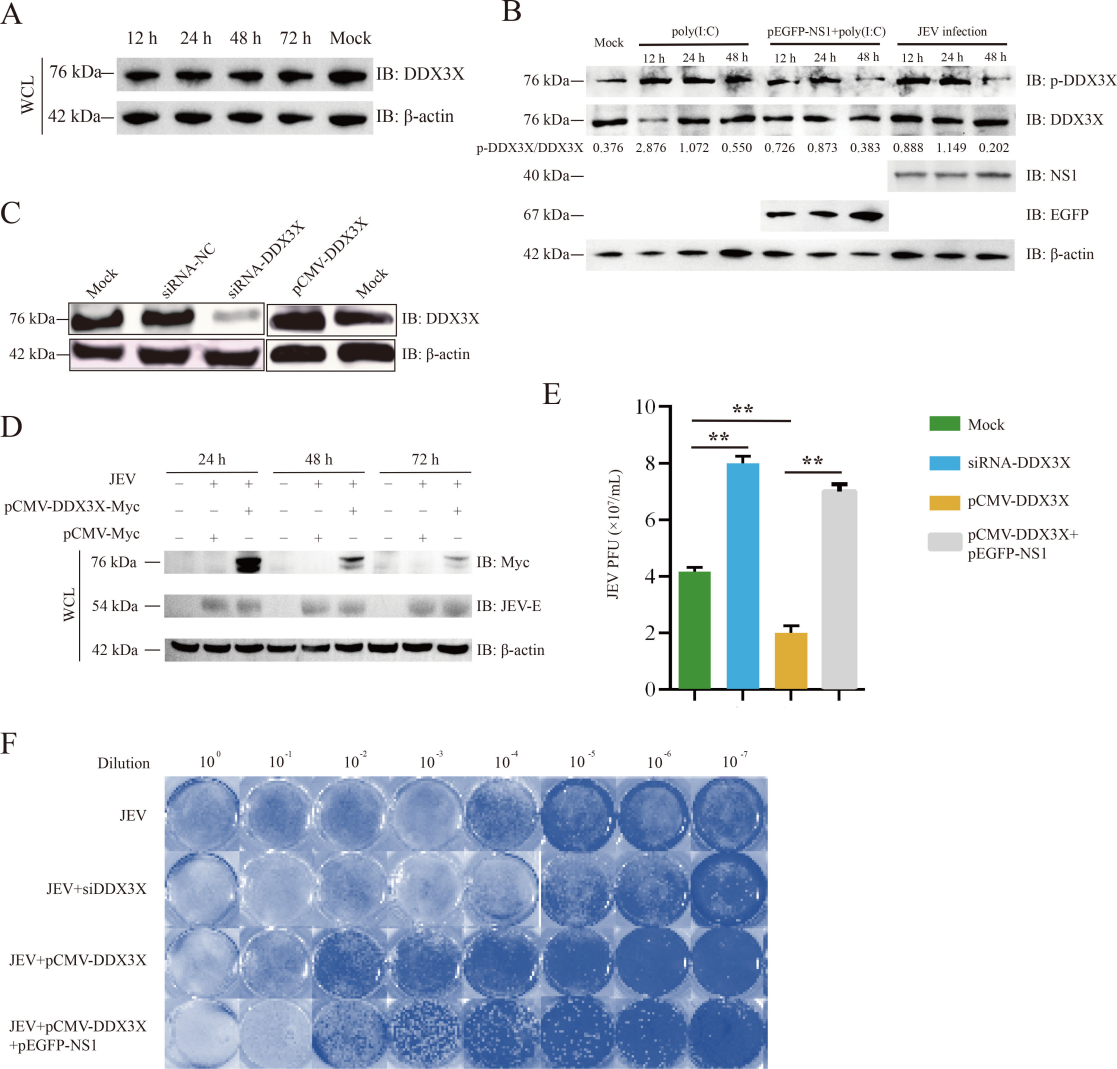
The influence of DDX3X on JEV replication. (**A**) BHK-21 cells were infected with JEV, and the whole cell lysates were collected at 12, 24, 48, and 72 h and then subjected to Western blot to detect DDX3X expression. (**B**) BHK-21 cells were transfected with pEGFP-NS1, co-treated with poly(I:C), treated with poly(I:C) alone, or infected with JEV (MOI = 0.1) to evaluate the phosphorylation of DDX3X. (**C**) BHK-21 cells were transfected with siRNA-DDX3X or pCMV-DDX3X, respectively, to ectopically express DDX3X or silence its expression. (**D**) BHK-21 was transfected with pMyc-DDX3X for 24 h and infected with JEV at an MOI = 0.1. Western blot was performed to detect DDX3X expression dynamics. (**E and F**) The JEV titers were detected by TCID_50_ and plaque assay. ,**P < 0.01.

NS1 is vital in viral genome replication and immune evasion (24). To analyze the biological significance of DDX3X-NS1 interaction, the expression level of DDX3X was first detected during JEV infection. As shown in Fig. 5A, JEV infection does not markedly influence the expression of DDX3X in BHK-21 cells. As a positive regulator of the RIG-I-like receptor pathway, DDX3X phosphorylation enhanced the activation of IRF3 (25). To determine the effects of NS1 on DDX3X phosphorylation, BHK-21 cells were transfected with pEGFP-NS1 and poly(I:C), treated with poly(I:C) alone, or infected with JEV (MOI = 0.1) to assess DDX3X phosphorylation. The results showed that poly(I:C) treatment could induce DDX3X phosphorylation, but this induction was inhibited when NS1 was overexpressed. A similar effect was observed during JEV infection (Fig. 5B). These findings suggest that NS1 may directly bind to DDX3X, preventing its phosphorylation and subsequently hindering the IRF3 signaling pathway.

Second, DDX3X was transiently overexpressed or knocked down in BHK-21 cells to determine its influence on JEV replication. BHK-21 was transfected with pMyc-DDX3X for 24 h and then infected with JEV at an MOI of 0.1. The whole cell lysates were collected and subjected to Western blot analysis using an anti-Myc antibody. The results indicated that the exogenous DDX3X expression peaked at 24 hpi, and thus, the time point was selected to determine the viral titers by TCID_50_ and plaque assay (Fig. 5D). The results showed that JEV replication was markedly inhibited in DDX3X-overexpressing cells (Fig. 5E and F). Then, we knocked down the expression of DDX3X by siRNA in BHK-21 cells (Fig. 5C). The viral titers at 24 hpi were determined, and the results demonstrated that DDX3X silencing markedly promotes JEV proliferation (Fig. 5E). A similar result was observed in the plaque assay, which showed that knocking down DDX3X promoted viral replication, while overexpression of NS1 inhibited this promotion. These findings demonstrate that DDX3X plays an anti-viral role in JEV infection, while the virus, in turn, utilizes NS1 to counteract DDX3X-induced anti-viral responses.

### The NS1-DDX3X interaction hinders IFN-β and ISG expression

**Fig 6:**
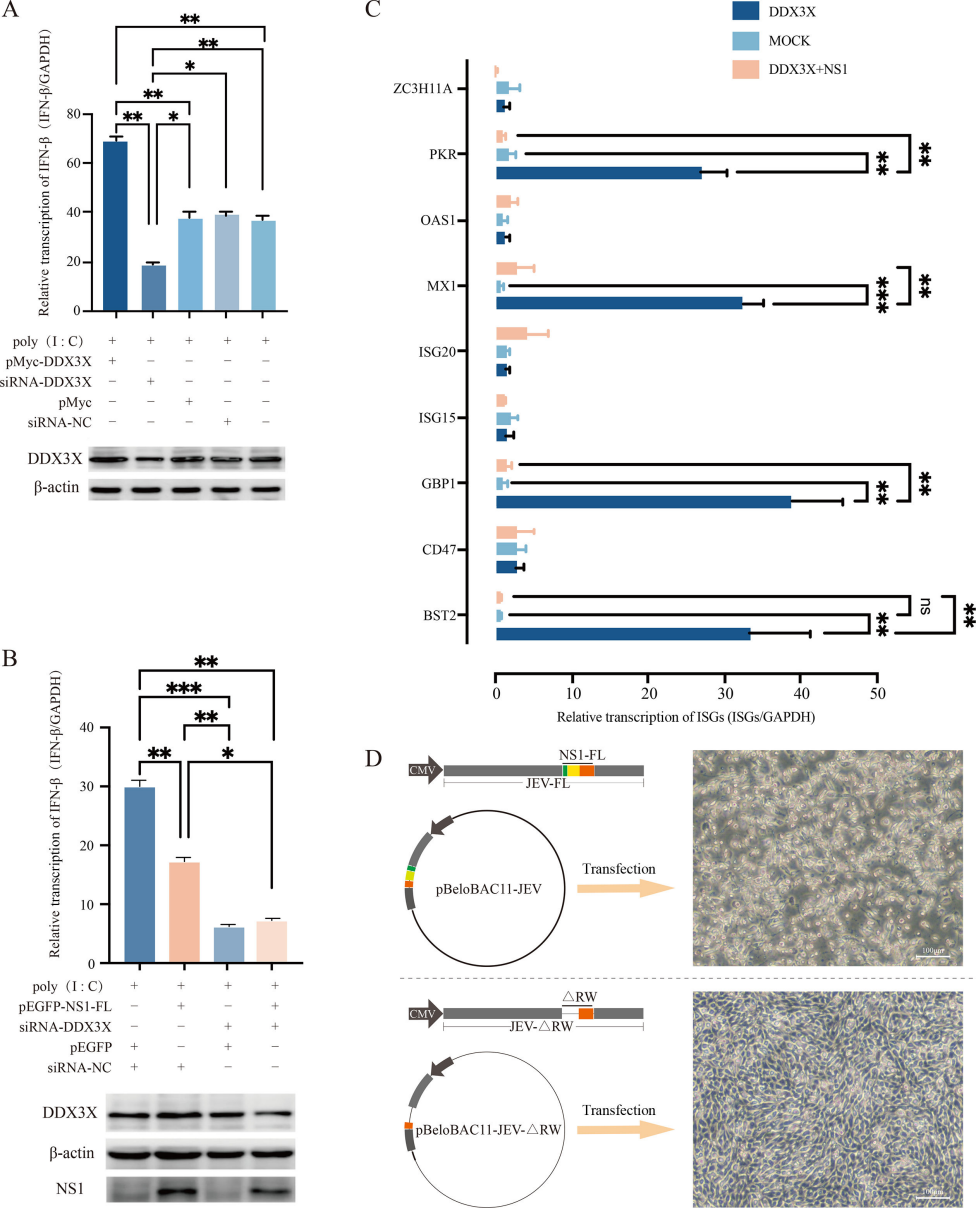
The NS1-DDX3X interaction affects IFN-β and ISG expression. (**A**) The effects of DDX3X overexpression or silencing on IFN-β transcription. (**B**) The effects of NS1 expression or DDX3X silencing on IFN-β transcription. (**C**) The effects of NS1 expression or DDX3X silencing on ISG transcription. *P < 0.05, **P < 0.01, ***P < 0.001. (**D**) JEV-FL and JEV-ΔRW (lacking the β-roll and wing domains) were constructed in the pBeloBAC11 vector using reverse genetics. These constructs were then transfected into BHK-21 cells for virus rescue, with the corresponding images showing BHK-21 cells (scale bar = 100 µm).

As an ATP-dependent RNA helicase, DDX3X was reported to be involved in the expression of IFN-β in DF-1 cells (26). First, the transcription of IFN-β was detected in DDX3X-overexpressed or DDX3X-silencing cells. The qRT-PCR results showed that the transcription of IFN-β was elevated in DDX3X ectopic expression cells but reduced in DDX3X-silencing cells (Fig. 6A). To further determine whether the NS1-DDX3X interaction inhibits JEV replication by affecting IFN-β production, the IFN-β transcription was quantified. The results showed NS1 markedly inhibited IFN-β transcription, and this effect was more notable in DDX3X-silencing cells (Fig. 6B).

IFN-β mainly binds to its receptor and induces the expression of ISGs to inhibit virus replication. Thus, the transcription levels of several ISGs were assessed by qRT-PCR in cells overexpressing DDX3X or DDX3X combined with NS1. The ISGs included were zinc finger CCCH-type containing 11A (ZC3H11A), protein kinase R (PKR), 2′,5′-oligoadenylate synthetase 1, myxovirus resistance 1 (MX1), ISG20, ISG15, guanylate-binding protein 1 (GBP1), cluster of differentiation 47, and bone marrow stromal antigen 2 (BST2). The results showed that DDX3X overexpression markedly induced the transcription of PKR, MX1, GBP1, and BST2, but this effect was hindered to a great extent by NS1, indicating NS1 may subvert the innate immune response by interacting with DDX3X to facilitate virus replication (Fig. 6C).

## DISCUSSION

The innate immune response, especially the production of IFN-β and the downstream ISGs, is the first line of defense against virus infection. Once the JEV enters the host cells, it replicates its genome, forming dsRNA intermediates in the cytoplasm and subsequently triggering the activation of RNA sensor RIG-I and melanoma differentiation-associated protein 5 (MDA5) (27). In addition, TLR3, located in the endosome, was also activated mainly during JEV entry (28). JEV has also evolved various countermeasures to escape the clearance of innate immune response by disrupting the actions of IFN and evading IFN-stimulated anti-viral responses: JEV escapes IFN production by STING degradation using NS2B3 protease (29); JEV antagonizes IFN-α signaling by preventing phosphorylation of Tyk2 and thus activation of the JAK-STAT signaling pathway (30); JEV NS2A hindered PKR activation (31). While the specific role of NS1 in JEV infection has not yet been fully elucidated, we aimed to investigate its function in greater detail. Through our studies, we found that NS1 inhibits IFN-β and ISG production by interacting with DDX3X, shedding light on its role in regulating the innate immune response.

Previous studies reported that NS1 was highly conserved and related to its pathogenesis in an unclear way (32, 33). Based on the mass spectrometry results, several host proteins were captured interacting with NS1 during JEV infection. DExD/H-box protein not only acts as PRRs to initiate innate immune response but also regulates the activation of PRRs downstream signaling pathways to affect the process of innate immune response (19). Therefore, the DExD/H-box proteins are hot points targeted by various viruses to participate in immune regulation (19). For example, DDX3 can interact with RIG-I, MDA5, and MAVS to enhance IFN promoter activity. In contrast, the innate immune response is weakened when DDX3 expression is knocked down (20). DDX3X could bind to the 5′ UTR and 3′ UTR of the viral genome to regulate viral replication when infected with JEV (34). In addition, DDX3X unwinds RNA in an ATP-dependent manner, suppresses DENV, and promotes WNV infection (22, 35). Thus, we speculate that NS1 may target DDX3X to escape the immune response. To confirm this hypothesis, the influence of DDX3X on JEV replication was studied in DDX3X overexpression or knocked-down cells, and the results indicated that DDX3X was an anti-viral factor during JEV infection. However, the mediators or signaling pathways that DDX3X hijacks to counteract JEV infection remain elusive.

**Fig 7:**
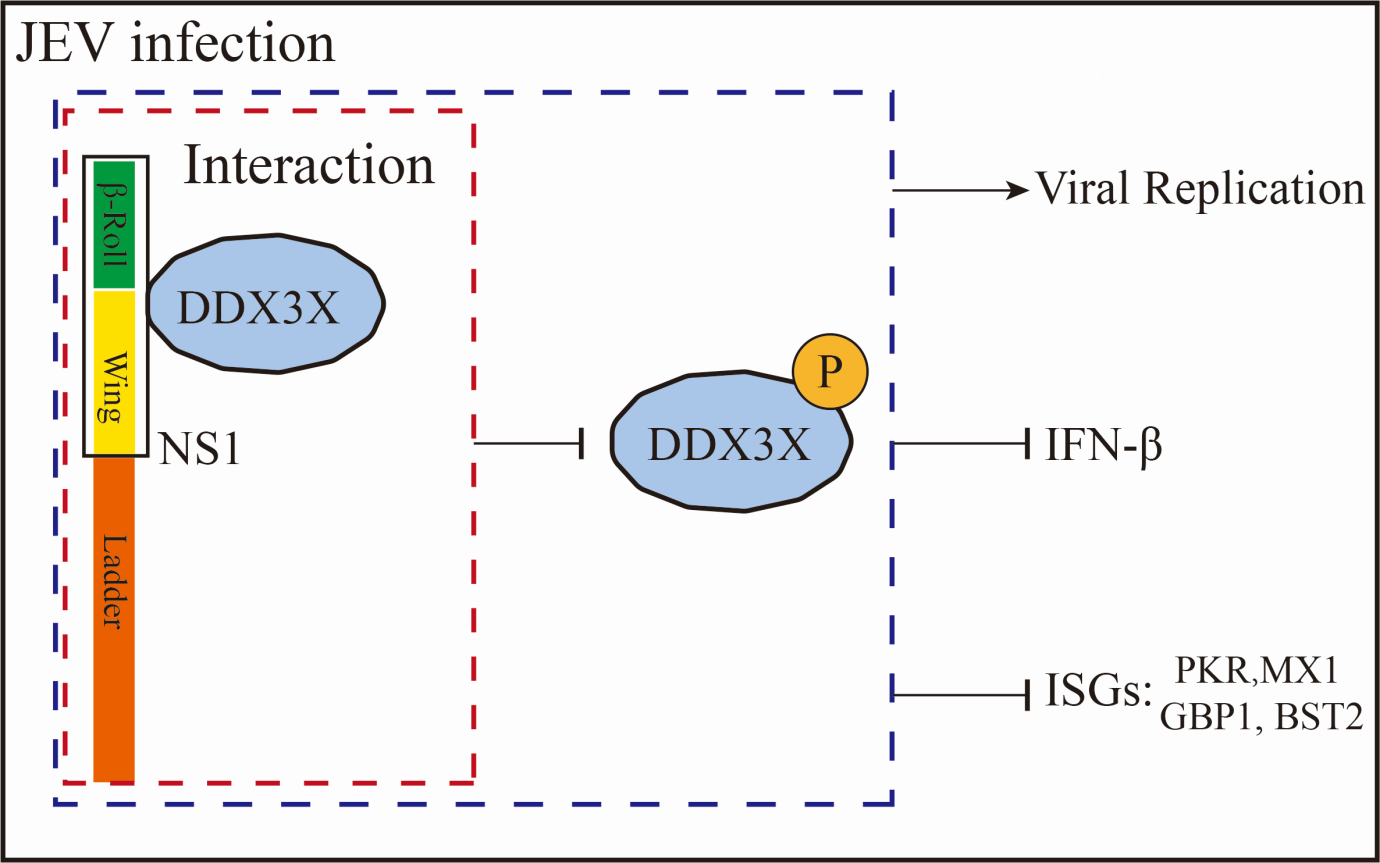
Interaction mechanisms of DDX3X and NS1. The diagram illustrates the role of DDX3X during JEV. DDX3X interacts with NS1 and becomes phosphorylated (**P**). The DDX3X-NS1 interaction promotes viral replication while suppressing the expression of interferon-β (IFN-β) and downstream ISGs, including PKR, MX1, GBP1, and BST2.

At the beginning of the study, the NS1-DDX3X interaction was confirmed *in vivo* and *in vitro*. Furthermore, the GST-pull-down assay result showed that NS1 binds to DDX3X directly (Fig. 3E). To further investigate the interaction domains, truncated mutants were generated, and a series of Co-IP analyses revealed that the amino acids from positions 1 to 180 of NS1 are critical for DDX3X binding (Fig. 4), and studies are required to elucidate the precise model of interaction. It should be noted that JEV infection induces DDX3X phosphorylation (Fig. 5B), and conversely, DDX3X phosphorylation can inhibit viral replication (Fig. 5E). Moreover, this inhibitory effect is diminished when DDX3X is co-expressed with NS1 (Fig. 5E). The Western blot and qPCR results, together with the plaque assays, suggest that NS1 interacts with DDX3X and participates in the signal pathways triggered by JEV infection. IFN-β is a key effector of the host’s innate immune response against viral infections. Figure 6A demonstrated that DDX3X is an IFN-β enhancer during virus infection. After IFN binding, JAK kinases associated with IFN receptors lead to downstream activation of the transcription factors STAT1 and STAT2 and induce the production of various ISGs, such as MX1, GBP1, and BST2. To explore the downstream signaling pathways of IFN, a random selection of ISGs was analyzed. The qRT-PCR data showed that DDX3X overexpression markedly elevated the transcription of PKR, MX1, GBP1, and BST2. In addition, exogenous expression of NS1 significantly dampened this effect, reducing it to a level comparable to that of the mock group. These data collectively indicate that DDX3X may inhibit JEV replication by inducing the expression of anti-viral ISGs, but NS1 could subvert this detrimental environment to evade host clearance. Figure 7 illustrates a potential mechanism involving the interaction between NS1 and DDX3X. Upon JEV infection, DDX3X plays a critical role in promoting IFN production as part of the host cell’s anti-viral response. However, NS1 binds to DDX3X, suppressing its anti-viral activity, thereby highlighting an intriguing interplay between the host’s defense mechanisms and the virus’s evasion strategies.

Based on the above observations, we planned to determine the critical amino acids and disrupt the NS1-DDX3X interaction. However, the β-roll and wing domains collectively mediate the NS1-DDX3X interaction, and truncation of β-roll and wing fails to rescue the virus (Fig. 6D). In this condition, small-molecule drugs capable of crossing the blood-brain barrier could be developed to block NS1-DDX3X interaction and inhibit JEV replication.

## SUPPLEMENTAL MATERIAL

Table S1 jvi.00077-25-s0001.docx Score table for host proteins interacting with the NS1 protein.

ASM does not own the copyrights to Supplemental Material that may be linked to, or accessed through, an article. The authors have granted ASM a non-exclusive, world-wide license to publish the Supplemental Material files. Please contact the corresponding author directly for reuse.
