# Supplementary material for: Japanese encephalitis virus NS1 inhibits IFN-β production by interacting with DDX3X
Source: J Virol. 2025 Apr 15;99(5):e00077-25. doi: 10.1128/jvi.00077-25 (PMC12090713; doi:10.1128/jvi.00077-25)
Supplement: Table S1 — Score table for host proteins interacting with the NS1 protein. [file jvi.00077-25-s0001.docx]

**TABLE S1** Score table for host proteins interacting with the NS1 protein

| Protein Name | Score |
| --- | --- |
| Vimentin (VIM) | 2989 |
| Heat shock protein 86 (HSP86) | 2489 |
| Heterogeneous nuclear ribonucleoprotein K (HnRPK1) | 1985 |
| L-lactate dehydrogenase A/B (LDHA/B) | 1876 |
| T-complex protein 1 subunit gamma (TCP-1-γ) | 1262 |
| 40S ribosomal protein S6（RPS6） | 1240 |
| Prefoldin (Prefoldin) | 499 |
| Ribosomal protein L4（RPL4） | 485 |
| Proteasome subunit alpha type (PSMA) | 482 |
| ATP-dependent RNA helicase DDX3X (DDX3X) | 112 |
